# Clinical profile and treatment outcomes of acute lymphoblastic leukemia among children attending at the University of Gondar comprehensive specialized hospital and Tikur Anbessa Specialized Hospital in Ethiopia

**DOI:** 10.1371/journal.pone.0322747

**Published:** 2025-06-05

**Authors:** Eyuel Kassa, Mulugeta Ayalew, Mastewal Birhan, Aschalew Gelaw, Abdulkader Mohammedsaid Gidey, Tadele Amare Zeleke, Degalem Tilahun Worku, Awol Mekonnen, Amare Kiflie, Ermias Teklehaimanot Yifter, Nega Berhane

**Affiliations:** 1 University of Gondar, Institute of Biotechnology, Department of Medical Biotechnology, Gondar, Ethiopia; 2 University of Gondar, College of Medicine and Health Sciences, School of Medicine, Department of Pediatrics and Child Health, Unit of Pediatric Hematology Oncology, Gondar, Ethiopia; 3 University of Gondar, College of Veterinary Medicine and Animal Sciences, Gondar Ethiopia; 4 University of Gondar, College of Medicine and Health Sciences, School of Biomedical and Laboratory Sciences, Department of Medical Microbiology, Gondar, Ethiopia; 5 Addis Ababa University, College of Medicine and Health Sciences, School of Medicine Department of Pediatrics and Child Health, Unit of Pediatric Hematology Oncology, Addis Ababa, Ethiopia; 6 University of Gondar, College of Medicine and Health Sciences, School of medicine, Department of psychiatry,; 7 Debre Berhan University, Asrat Woldeyes Health Science Campus, Department of Pharmacy,; 8 University of Gondar, College of Medicine and Health Sciences, Department of pathology; Debre Markos University, ETHIOPIA

## Abstract

**Background:**

Acute lymphoblastic leukemia (ALL) is a common childhood cancer characterized by the uncontrolled proliferation of immature white blood cells. While advancements in treatment have significantly improved outcomes in developed countries, significant challenges remain in resource-limited settings, such as Ethiopia. This study aimed to assess the clinical profiles and treatment outcomes of ALL patients at the University of Gondar Comprehensive Specialized Hospital (UoGCSH) and Tikur Anbessa Specialized Hospital (TASH) in Ethiopia.

A prospective longitudinal study was conducted among 179 ALL patients receiving treatment at the outpatient department and pediatric oncology centers of the UoGCSH and TASH between December 25, 2022, and August 30, 2024. Sociodemographic and clinical data were collected using a structured questionnaire. The data were entered and analyzed using SPSS version 25. Descriptive statistics were employed to summarize patient characteristics, while overall survival was evaluated using Kaplan-Meier analysis. Additionally, both univariate and multivariate Cox proportional hazards regression analyses were performed, with statistical significance set at a *P*-value of < 0.05.

**Results:**

Among the 179 patients, 81 (45.3%) died during the course of treatment. Of these, 33 (18%) died before initiating induction therapy, while 48 (27.4%) died primarily due to treatment abandonment during various phases of therapy. The event-free survival rate was 75 (41.9%). Mortality rates were significantly higher in patients with certain variables identified through Cox regression analysis, including age, sepsis, and relapse, which nearly doubled the risk of death. Elevated LDH levels, malaria infection, and T-cell ALL were associated with approximately six-fold, three-fold, and seven-fold increases in the risk of death, respectively. Only 22 out of 179 patients (12.29%) achieved remission. Among these patients, hematotoxicities observed during the maintenance phase included anemia in 19/22 (86.4%), grade 3–4 neutropenia in 12/22 (52.2%), and thrombocytopenia in 17/22 (77.3%).

**Conclusion:**

A high mortality rate was observed among children with ALL, with significant risk factors including relapse, age over 10 years, elevated LDH levels, sepsis, low platelet counts, T-cell ALL, malaria infection, and induction failure. To improve survival rates, it is essential to address these factors by optimizing treatment regimens and minimizing delays in diagnosis and care delivery.

## Introduction

Leukemia is the most common malignancy in children, accounting for approximately 30% of all cancers diagnosed in children under 15 years of age in developed countries [1]. Globally, acute lymphoblastic leukemia (ALL) represents the most prevalent pediatric cancer, characterized as a malignancy of B or T lymphocytes involving the uncontrolled proliferation of abnormal, immature lymphocytes. Worldwide, nearly 400,000 new cases of childhood cancer (age range, 0–19 years) are reported annually, with low- and middle-income countries (LMICs) accounting for a significant proportion (90%) of these cases [2]. However, for much of the past three decades, leukemia’s were considered rare hematological cancers, with only sporadic cases reported, particularly in regions like Africa, where data were scarce and the condition was often thought to be nonexistent [3]. In Ethiopia, the annual incidence of childhood cancers is estimated to be between 3,707 and 6,000 cases, with leukemia being the most prevalent, accounting for 29% of these cases. Acute leukemia constitutes 89% of all childhood leukemia cases in Ethiopia, with ALL representing 91% and acute myeloid leukemia (AML) accounting for the remaining 9% [4,5].

In high-income countries (HICs), survival rates have drastically improved from 30% to 90% over the past 50 years. A recent report by the children’s oncology group stated that the overall survival rate for children with standard-risk B-cell ALL is approximately 96% [1,6–9]. However, 30–40% of these patients relapse during the maintenance phase. The relapse rate in developed countries has been 11% in recent years. In contrast, survival rates in LMICs, remain poor, often around 35%. Mortality rates for most pediatric cancers are close to 100% in developing countries, including Ethiopia [10]. Treatment abandonment is a major reason for treatment failure and low survival rates in LMICs [11], and this issue is directly correlated with the country’s income level [12]. The stark disparity in pediatric cancer survival rates between HICs and LMICs is largely attributable to differences in healthcare systems. Inadequate government health spending in LMICs often results in inefficient healthcare delivery, contributing to several factors that undermine survival rates. These factors include delayed diagnosis, insufficient numbers of physicians and nurses, limited supportive care infrastructure, restricted access to effective treatments, high treatment-related mortality rates, increased relapse rates, and elevated rates of treatment abandonment [13].

The treatment regimen for childhood ALL typically involves the induction of remission through chemotherapy at the time of diagnosis, followed by consolidation therapy, delayed intensification, and maintenance therapy [14]. Over the years, treatment protocols for ALL have improved significantly evolving from aminopterin, which achieved only a temporary remission in 1948 [15], to the chemotherapy regimens used today. These regimens have been refined through the consolidative clinical experiences of the Italian Association of Pediatric Hematology (AIEOP) and Berlin-Frankfurt-Münster Study Group (BFM) study groups. As a result of these improved protocols, the current treatment for ALL now achieves a 5-year survival rate of 92% [16]. Thiopurines, such as 6-mercaptopurine (6-MP), are critical components of the current ALL treatment. However, their use is associated with significant toxicity, particularly myelosuppression, due to interindividual variability in thiopurine S-methyltransferase (TPMT) enzyme activity [17–19].

Baseline data on diseases are essential for understanding disease patterns, treatment outcomes, and informing effective public health policies. By studying ALL, researchers can identify trends, develop optimal treatment strategies, and advocate for improved resource allocation and public awareness. Consequently, this study aims to address a critical gap by providing a detailed analysis of the clinical characteristics, treatment outcomes, and factors influencing survival in pediatric ALL patients treated at two major referral hospitals in Ethiopia. By identifying key barriers to optimal care and predictors of poor outcomes, this research seeks to inform targeted interventions and policy changes to improve survival rates and the quality of care for children with ALL in resource-limited settings. Furthermore, the findings will contribute to the broader understanding of ALL management in Ethiopia and other LMICs, supporting global efforts to reduce disparities in childhood cancer outcomes.

## 2. Materials and methods

### 2.1. Study area and setting

The study was conducted at the UoGCSH and TASH, in Ethiopia. The University of Gondar Hospital is a referral hospital located 727 km from the capital, Addis Ababa, in the northwestern part of the country. The hospital has a dedicated pediatric hematology-oncology unit with 35 beds, as well as a separate adult oncology unit. Currently, it serves approximately seven million people, providing both outpatient and inpatient services. As a referral center, it offers various medical services to the surrounding areas and neighboring regions, including adult and pediatric hematology-oncology care. The pediatric hematology-oncology center at UoGCSH is the only one in the region, with essential imaging and pathology services available. Tikur Anbessa Specialized Hospital, located in Addis Ababa, serves as a teaching hospital for the country’s leading medical and health Sciences University. It is the first and largest hospital in Ethiopia to offer hematology-oncology services. Together, both hospitals serve more than 10 million people within their catchment areas [10,20–22].

### 2.2. Study design and period

An institution-based prospective longitudinal study design was used to evaluate the clinical profile and treatment outcomes of children with ALL at the UoGCSH and TASH from December 25, 2022, to August 30, 2024.

### 2.3. Source and study population

The source population included all children who visited the pediatrics OPD, as well as those admitted to the pediatric oncology unit during the study period at the UoGCSH and TASH. All children below the age of 18 years who were diagnosed with ALL were the study population.

### 2.4. Eligibility

#### 2.4.1. Inclusion criteria.

All patients below the age of 18 who presented for care, were diagnosed with ALL and were admitted to UoGCSH and TASH from December 25, 2022, to August 30, 2024. All patients had a confirmed pathologic diagnosis of ALL based on biopsy samples.

#### 2.4.2. Exclusion criteria.

Patients suspected of having ALL based on clinically findings and FNAC results, but without a confirmed diagnosis from biopsy pathology, were excluded. Additionally, patients without a confirmed diagnosis of ALL or those who did not provide consent were also excluded from the study.

### 2.5. Operational definition

- **Treatment outcome:** The result obtained after the use of a drug. Which may include cure, death, or complication.
- **Neutropenic fever:** A temperature ≥ 100.4F (38.3°C) for at least an hour, accompanied by an absolute neutrophilic count (ANC) of less than 1500cells/µL.
- **Complete Remission**: Defined as the eradication of all detectable leukemia cells (less than 5 percent blasts) from the bone marrow and blood, along with the restoration of normal hematopoiesis (>25 percent cellularity and normal peripheral blood counts).
- **Relapse:** The reappearance of leukemia cells in the bone marrow or peripheral blood after the attainment of complete remission [23].
- **CNS Involvement: **≥ 5 cells/mm3 of CSF analysis [24].

### 2.6. Risk stratification and treatment protocols

The treatment regimen for ALL was determined based on patient stratification into standard risk (SR) and high-risk (HR) categories. The SR ALL is defined as patients aged 1 to less than 10 years with a white blood cell count below 50,000/µL, no CNS blast cell infiltration at presentation, and confirmed bone marrow remission. The SR ALL patients received treatment in three phases: induction, consolidation, and maintenance [25]. The HR patients, in this group underwent additional treatment phases, such as re-induction or intensification, before entering the maintenance phase. A four-drug regimen consisting of prednisolone, vincristine, L-asparaginase, and doxorubicin was administered for most patients, including HR patients with central nervous system (CNS) involvement. For patients with CNS involvement, hydrocortisone and intrathecal cytarabine were added to the treatment regimen. In SR patients, however, doxorubicin was omitted from the treatment regimen. The specific timelines for each phase of treatment may differ depending on individual risk factors and the treatment protocols employed. Initially, a clinical and hematological assessment is performed, followed by induction therapy for pediatric ALL, which generally lasts between 4–8 weeks with the goal of achieving remission. This is succeeded by a consolidation phase that lasts approximately 6 months, aimed at eradicating any remaining leukemia cells.

### 2.7. Data collection tools and laboratory analysis

A structured questionnaire was used to collect demographic information, while a review of medical records was conducted to gather data on diagnosis, treatment protocols, therapeutic response, and follow-up information. A 4 ml venous blood sample was obtained from each patient for complete blood count (CBC), and morphology assessment. The CBC was performed using Sysmex XS-500i and XT-1800 analyzers (Sysmex Corporation, Kobe, Japan). Wright’s stain was performed for peripheral morphology assessment. Subsequently, peripheral morphology and bone marrow aspiration examinations were performed by an experienced pathologist. Following the conclusion of the six-month maintenance phase, clinical and hematological evaluations were conducted. Consequently, patients were monitored for a minimum duration of 13 months (1 year and 1 month).

### 2.8. Quality assurance mechanisms

Data on the sociodemographic characteristics of children and their caregivers, along with related factors, were collected through face-to-face interviews using a structured questionnaire adapted from various sources. Three BSc nurses conducted data collection under the close supervision of two pediatric hematology-oncology fellows at both study sites. Data collectors and supervisors received two days of training on study objectives, data collection tools, techniques, and ethical considerations. Interviewers manually recorded responses to both closed and open-ended questions. Supervisors assessed daily data consistency and completeness.

To ensure data quality, the questionnaire was adapted from previous studies [26,27] and reviewed by oncology experts. It was subsequently translated from English to Amharic by language experts. Rigorous quality control measures were implemented for laboratory procedures, including regular maintenance and cleaning of equipment to ensure accurate and reliable results. Additionally, data quality was validated using statistical parameters to assess the reliability and validity of the collected data.

### 2.9. Statistical analysis

Data were entered into Epi-Info version 7 and analyzed using SPSS version 25. Descriptive statistics, including frequencies, means with ± standard deviations, and medians with interquartile ranges, were used to summarize sociodemographic, clinical, and laboratory characteristics. Survival analysis was performed using the Kaplan-Meier method, and the log-rank test was used to compare survival rates. Univariate and multivariate Cox proportional hazard regression models were fitted to identify factors associated with survival in ALL. Statistical significance was determined at the *p* < 0.05 level with a 95% confidence interval.

### 2.10. Ethical considerations

Ethical clearance was obtained from the University of Gondar Institutional Ethical Review Board (Rfe. VP/RTT/05/246/2022), and a supportive letter was provided by TASH, Health Science College, Addis Ababa University. Every effort was made to protect participants’ well-being and dignity. Voluntary participation was emphasized, and participants had the right to withdraw from the study at any time. Informed consent was obtained from caregivers, and assent was obtained from children as appropriate. All research procedures adhered to the Declaration of Helsinki.

## 3. Results

### 3.1. Socio-demographic and clinical characteristics of study participants

A total of 179 children with ALL were enrolled in the study. Of these, 115 (64.2%) were male. The age of the participants ranged from 1 to17 year, with a mean age of 8.06 ± 4.05 years. The majority of participants, 122(68.2%) were aged 1–10 years, followed by those aged 10–17 years. More than half the participants, 95 (53.1%) were rural residents, and 135 (75.5%) of the caregivers were unemployed. Most caregivers reported a monthly income below 1,000 ETB. The regional distribution of the study participants was as follows: 105 (58.66%) of the patients were from Amhara, 35 (19.55%) from Oromiya, 16 (8.94%) from the Southern Nations, Nationalities, and Peoples Region (SNNP), 15 (8.38%) from Addis Ababa, 5 (2.79%) from Somalia and 3 (1.68%) from Tigray (Table 1).

**Table 1:** Sociodemographic characteristics of study participants and caregivers of pediatric ALL patients at the UoGCSH, and TASH, Northwestern Ethiopia, 2024 (n = 179).

Variables	Category	Frequency	Percentage (%)
Gender	Female	64	35.8
	Male	115	64.2
Age range in years	>10	55	30.7
	1-10	122	68.2
	<1	2	1.1
Residence	Rural	95	53.1
	Urban	84	46.9
Marital status of the caregivers	Single	11	6.1
	Married	158	88.3
	Divorced	9	5.0
	Widowed	1	0.6
The monthly income of the caregiver	No regular monthly income	56	31.3
	less than 1000	14	7.8
	1000-3000	33	18.4
	3001-5000	40	22.3
	5001−10,000	26	14.5
	Greater than 10,000	10	5.6
Study setting	UoGCSH	90	50.1
	TASH	89	49.9
Regional distribution of patients	Amhara	105	58.66
	Oromia	35	19.55
	South Nations, Nationalities and people	16	8.94
	Addis Ababa	15	8.38
	Somalia	5	2.8
	Tigray	3	1.7

### 3.2. Initial clinical presentation of children diagnosed with ALL

The most common presenting symptom was bone pain observed in 154 (86.0%) of the patients. Neutropenic fever was the second most common symptoms, affecting 146 (81.6%) patients; however, osteonecrosis was not found in these study participants. Persistent infection was observed in 134 (74.9%) of the patients. Loss of appetite was noted in 153 (85.5) patients and behavioral alteration was observed in 128 (71.5%) patients. Petechiae (skin rash) occurred in 108 (60.3%) patients. Anemia was present in 144 (80.4%) patients, and abdominal swelling was noted in 84 (46.9%). The most common laboratory finding was severe anemia, affecting 22.9% of the patients (hemoglobin < 7 g/dl). Regarding physical signs, pallor was the most common, observed in 145 (81.0%) of the patients. Lymphadenopathy (LAP) was detected in 26 (14.5%), and subcutaneous nodules were found in 10 (5.6%) of the patients. Additionally, 7 (3.9%) patients exhibited raddish discoloration of the urine, and 5 (2.8%) had eye involvement. Regarding the ALL status of the children, 92 (51.4%) were diagnosed with SR ALL, and 87 (48.6%) with HR ALL. Of these, 146 (81.6%) were alive, while 33 (18.4%) had passed away before treatment initiation during the study period at the two study settings (Table 2).

**Table 2:** Initial clinical characteristics and hematological Profile of children with ALL at presentation at the UoGCSH and TASH, Northwestern Ethiopia 2024 (n = 179).

Variables N=	Category	Frequency	percentage
Presenting signs and symptoms	Neutropenic fever	146	81.6
	Behavioral alteration	128	71.5
	Loss of appetite	153	85.5
	Fatigue	153	85.5
	Bone pain	154	86.0
	Pallor	145	81.0
	Persistent infection	134	74.9
	Petechiae (skin rash)	108	60.3
	Abdominal swelling	84	46.9
	Malaise	64	35.8
	Lymphadenopathy	26	14.5
	Subcutaneous nodule	10	5.6
	Persistent headache	9	5.0
	Reddish discoloration of urine	7	3.9
	Reddish discoloration of the eye	5	2.8
ALL risk status	Standard risk	92	51.4
	High risk	87	48.6
ALL type	B cell ALL	17	9.5
	T cell ALL	13	7.3
	Unclassified ALL	149	83.2
French-American-British (FAB) classification	L1	68	38
	L2	86	48
	L3	14	7.8
	Non categorized ALL	11	6.1
CNS involvement	Yes	18	10.1
	No	161	89.9
Thrombocytopenia	<150 x10^9^/L	135	75.4
	≥150 x x10^9^/L	44	26.4
Anemia	Yes	144	80.4
	No	35	19.6
Malaria positive	Yes	22	12.3
	No	157	87.7
Blood transfusion	Yes	130	72.6
	No	49	27.4
Weight loss	Yes	123	68.7
	No	56	31.3
Mortality outcome before induction treatments	Alive	146	81.6
	Died	33	18.4

### 3.3. Changes in hematologic parameters across treatment phases

Initial white blood cell (WBC) counts ranged from 1.10 x 10^3^ counts/mm³ to 465 x 10^3^ counts/mm³, hemoglobin (Hgb) levels ranged from 1.60 g/dl to 31.70 g/dl, and platelet counts ranged from 2.0 counts/mm^3^ to 700,000 counts/mm^3^. The initial median white blood cell count, hemoglobin level, and platelet count at presentation were 42,000 counts/mm³ (IQR: 72,200 counts/mm³), 8.9 g/dl (IQR: 3.50 g/dl), and 97 (IQR: 170/mm³), respectively. Overall, the hematological profile at the end of consolidation indicates persistent cytopenias, particularly leukopenia, anemia, and thrombocytopenia. During the third phase, significant hematological challenges were faced by children with ALL undergoing mercaptopurine maintenance therapy. The high prevalence of low white blood cell counts (15/22, 68.2%), low hemoglobin levels (7–10 g/dl) (12/22, 54.5%), and platelet counts < 20k (6/22, 27.3%) were notable (Table 3).

**Table 3:** Changes in hematologic parameters across treatment phases at the UoGCSH and TASH, Northwest Ethiopia 2024 (n = 179).

Variables		Category	Frequency (%)	Median(IQR) & initial WBC range
Pre-treatment hematological profile **(n = 179)**	White blood cell count	<4x 10^3^	21 (11.7)	42 x 10^3^ (6.97-80x10^3^) 1.10 to 465 x10^3^counts/mm^3^
		4-49.9 x10^3^	80 (44.7)	
		≥50 x10^3^	78 (43.6)	
	Hemoglobin	<7 g/dl	41 (22.9)	6.93 g/dl (6.9–10.4 g/dl) 1.60 to 31.70 g/dl
			7-10 g/dl	92 (51.4)
			>10 g/dl	46 (25.7)
	Platelet count	<20k	51 (28.5)	89.96x 10^3^ (28.8–149.1 x10^3^) 2.0 to 700,000 counts/mm^3^
			20- 150k	84 (46.9)
			>150k	44 (24.6)
Induction phase (n = 120/179)	White blood cell count	<4x 10^3^	18 (15.0)	6.0 x 10^3^counts (4.65–11.4 x 10^3^counts)
		4-11 x10^3^	72 (60.0)	
		11-49x10^3^	22 (18.3)	
		≥50 x10^3^	8 (6.7)	
	Hemoglobin level	<7 g/dl	11 (5.2)	9.91 g/dl(8.0–11.03 g/dl)
			7-10 g/dl	57 (26.9)
			>10 g/dl	52 (24.5)
	Platelet count	<20k	12 (10.0)	160.3 x 10^3^ (120–253 x10^3^)
			20-150k	39 (32.5)
			>150	69 (57.5)
**Consolidation Phase (N = 107/179)**	White blood cell count	<4x 10^3^	59 (55.1)	3.55x 10^3^counts/mm^3^ (2.36–6.35 x 10^3^counts/mm^3^)
		4-11 x10^3^	44 (41.1)	
		11-49 x10^3^	4 (3.7)	
	Hemoglobin level	<7 g/dl	33 (30.8)	10.75 g/dl (10.01–11.73 g/dl)
			7-10 g/dl	49 (45.8)
			>10 g/dl	25 (23.4)
	Platelet count	<20k	25 (25.2)	143 x 10^3^counts/mm^3^ (59.0–170.0 x10^3^counts/mm^3^)
			20-150k	62 (57.9)
			>150	20 (18.7)
**Maintenance phase (N = 22/179)**	White blood cell count	<4x 10^3^	15 (68.2)	3.15x 10^3^counts/mm^3^ (2.07–4.69x10^3^counts/mm3)
		4-11 x10^3^	5 (22.7)	
		≥11x10^3^	2 (9.1)	
	Hemoglobin level	<7 g/dl	8 (36.4)	7.25g/dl (4.6-9.4g/dl)
			7-10 g/dl	12 (54.5)
			>10 g/dl	2 (9.1)
	platelet count	<20k	6 (27.3)	96 x 10^3^counts (18.4–138.5 x 10^3^counts/mm^3^)
			20- 150k	12 (54.5)
			>150k	4 (18.2)

At the end of the induction phase, the median white blood cell (WBC) count was 12,000/mm³, indicating an improvement compared to the initial hematological profiles. The majority of patients, 85(70.8%), gained weight during induction therapy. A small percentage of patients, 9 (7.5%), experienced treatment failure during induction therapy, while 18 (10.1%) of ALL patients relapsed at different phases of treatment (Table 4).

**Table 4:** Clinical characteristics of ALL patients following the induction period.

Variables	Category	Frequency	Percentage
Weight gain (end of induction)	Yes	85	70.8
	No	35	29.2
Induction failure	Yes	9	7.5
	No	111	92.5
Relapsed ALL	Yes	18	10.1
	No	161	89.9

### 3.4. Chemotherapy-related side effects during the first six months of maintenance therapy

Among the adverse effects and complications associated with treatment, hepatotoxicity, characterized by elevated liver transaminases, was observed in 9 (40.9%) of patients, predominantly during the maintenance phase. The most frequently reported side effects were fever and flu-like symptoms, which occurred in 18 (81.8%) patients. Nausea and vomiting were reported in 17 (77.3%) patients, itching or skin rash in 15 (68.2%) patients, and loss of appetite in 11 patients (50%), making them the most common side effects consecutively. Furthermore, mucositis was reported in 7 patients (31.8%), neutropenic fever affected 18 patients (81.8%). A significant proportion of patients experienced hematological side effects such as neutropenia 12(52.2%), leukopenia 15(68.2%), thrombocytopenia 17(77.3%), and anemia 19(86.4%). These are common consequences of chemotherapy. These side effects are common outcomes of chemotherapy and can considerably affect the quality of life for patients as well as their adherence to treatment. Most of the study participants who completed their treatment were male (13/22, 59.1%) and urban residents (16/22, 72.7%) (Table 5).

**Table 5:** Incidence of chemotherapy-related side effects during the first 6 months of maintenance therapy (N = 22/179).

Variables	Categories	Number (n)	Percentage (%)
Gender	Male	13	59.1
Residents	Female	9	40.9
	Urban	16	72.7
	Rural	6	27.3
Clinical characteristics	Neutropenia grade 3 & 4	12	52.2%
	Sepsis	6	27.3
	Mucosytosis	7	31.8
	Hepatotoxicity	9	40.9
	Leukopenia	15	68.2
	Thrombocytopenia (< 150 x 10^9^/L)	17	77.3
	Neutropenic fever	18	81.8
	Anemia (<11.5 g/dl)	19	86.4
	Treatment interruption	12	54.5
	Flu-like symptoms	18	81.8
	Itching or skin rash	15	68.2
	Hair loss	9	40.9
	Loss of appetite	11	50
	Nausea/vomiting	17	77.3
	Behavioral alteration	14	63.6
	Total mortality (n = 179)	81	45.8

### 3.5. Treatment Outcomes and Abandonment Rates in ALL Patients Across Induction, Consolidation, and Maintenance Phases

**Fig 1:**
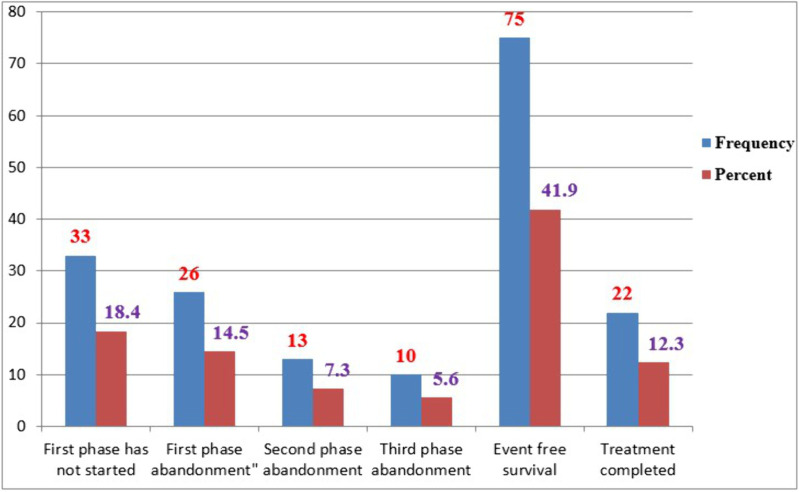
Treatment outcomes of pediatric ALL patients across different treatment phases at the UoGCSH and TASH, Northwestern Ethiopia, 2024, (n = 179).

The rates of treatment abandonment during the induction, consolidation, and maintenance phases were 26 (14.53%), 13 (7.3%), and 10 (5.6%), respectively. Additionally, 75 (41.9%) of the study participants achieved event-free survival. Across all treatment phases, 22 (12.29%) of patients completed their treatment (Fig 1). Treatment abandonment was significantly higher among children from rural backgrounds (p < 0.001), those with lower socioeconomic status (p < 0.001), and those with low hemoglobin levels (p = 0.000) or severe wasting (p = 0.001).

### 3.6. Kaplan-Meier analysis of overall survival in ALL patients

Kaplan-Meier survival curves were generated to assess the impact of key prognostic factors on overall survival in patients with ALL. It was hypothesized that patients presenting with elevated WBC counts would exhibit a steeper decline in the survival curve during the initial period, reflecting an increased risk of early mortality. This is consistent with the established association between high initial WBC counts and a more aggressive disease phenotype. Similarly, patients with concomitant malaria infection were expected to demonstrate a significantly steeper decline in survival compared to malaria-negative patients, suggesting a poorer prognosis. Furthermore, CNS involvement at diagnosis was also anticipated to correlate with a steeper survival curve, indicative of a diminished survival probability.

**Fig 2:**
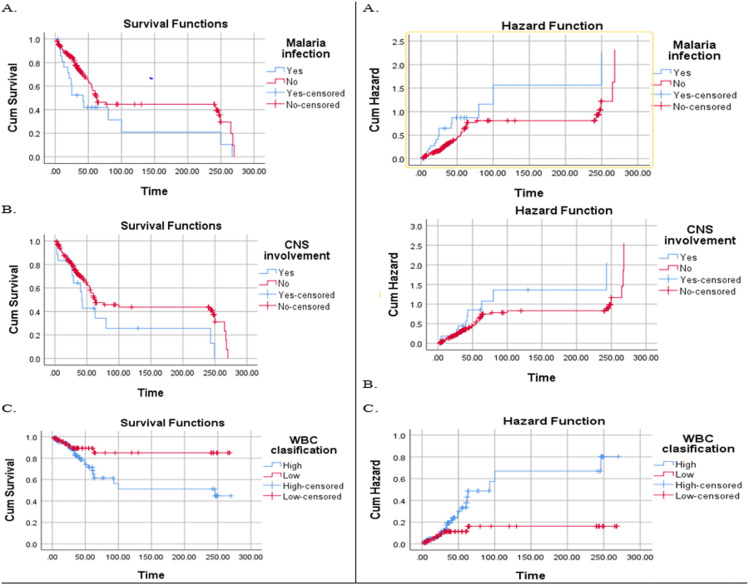
Kaplan–Meier survival curves of the patients with ALL at the UoGCSH and TASH.

Kaplan-Meier hazard curves demonstrated a significantly elevated cumulative risk of mortality in patients with high initial WBC counts, malaria infection, and CNS involvement, compared to those with low initial WBC counts, absence of malaria, and no CNS involvement (P < 0.05) (Fig 2).

### 3.7. Predictor of for mortality during the treatment phase

Cox proportional hazards regression analysis was performed to identify predictors of mortality during the treatment phase of ALL. The univariate analysis revealed that an initial leukocyte count of ≥50x10^3^/µL (p > 0.05), sex (p > 0.05), and CNS involvement (p < 0.058) did not show statistical significance. In contrast, factors such as platelet count (p < 0.002), age (p < 0.011), relapsed ALL patients (p < 0.001), sepsis (p < 0.047), LDH levels (p < 0.008), malaria infection (p < 0.043), and area of residence (p < 0.050) were significantly associated with mortality. The multivariate analysis identified several statistically significant variables: the absence of bone marrow remission after the induction phase (relapsed) increased the risk nearly twofold; age over 10 years increased the risk by 2.2 times; elevated LDH levels raised the risk by 2.7 times; sepsis increased the risk by 2.4 times; low platelet counts nearly doubled the risk; and T-cell ALL, malaria, and induction failure were associated with approximately sevenfold, fourfold, and fourfold higher risks of death, respectively (Table 6).

**Table 6:** Cox-regression of risk factors for mortality during the treatment phase at the UoGCSH and TASH.

Variables	Categories	Mortality treatments outcome (n = 179)		Univariate Hazard Ratio (95% CI)	Multivariate Hazard Ratio (95% CI)
		Alive (n = 98)	Died (n = 81)		
Sex of the child	Female	34	30	1	
	Male	64	51	0.852(.562 −1.294)	0.615(0.285-1.325)
Age of the child	>10 year	25	30	1	
	< 10 year	73	51	2.177(1.349-3.512)	2.240(1.345-3.730)
Area of residence	Rular	54	41	2	
	Urban	44	40	1.160 (.778–1.730)	0.987(0.435-2.239)
Induction failure	Yes	7	2	1	
	No	91	79	2.267(1.039-4.944)	4.741(.600-37.493)
CNS	Yes	5	13	1	
	No	93	68	2.156(0.876-5.306)	0.105(0.019−.572)
Sepsis	Yes	9	16	1	
	No	89	65	2.238(1.112-4.506	2.475(1.053-5.817)
LDH, (n = 89)	Yes	32	30	1	
	No	10	17	2.888(1.33-16.263)	2.735(1.505-4.970)
Platelets	<20k	25	26	1	
	20-150k	46	38	1.163 (1.7121-0.901)	1.240(0.752-2.042)
	> 150k	27	17	1.328(0.769-2.294)	1.959(1.060-3.619)
WBC count	High	58	51	2	
	Low	40	30	0.920(0.612-1.382)	0.328(0.106-1.012)
Malaria infection	Yes	6	16	1	
	No	92	65	0.687(0.377 - 1.254)	4.24(1.499-36.037)
Relapsed	Yes	6	12	1	
	No	92	69	1.513(0.62-3.458	1.533(0.656-3.586)
ALL HR & SR	SR	55	37	2	
	HR	43	44	1.198(0.803 −1.789)	0.741(0.338-1.625)
Type of ALL	B cell	13	4	1	
	T cell	3	19	3.358(1.049-10.747)	7.483(1.999-28.014)
	unclassified	82	67	2.327(.845 −6.411)	3.314(0.975-11.266)

## Discussion

The significant improvement in survival rates for childhood ALL represents a major milestone in cancer treatment. In the 1960s, only 15% of children diagnosed with ALL survived for five years post-diagnosis. Today, over 80% of these children achieve five years, with a remarkable 93.5% considered cured [28]. Recent advancements have enabled nearly all children with standard-risk ALL (100%) to achieve a cure [29,30]. However, treatment outcomes for ALL in sub-Saharan Africa remain suboptimal, facing substantial challenges [31]. To our knowledge, this is the first prospective longitudinal study to describes the clinical profile and treatment outcomes for ALL among pediatric patients in Ethiopia and more broadly, across sub-Saharan Africa.

The most common initial clinical feature among patients with ALL was anemia, observed in 144 patients (80.4%). These findings are consistent with a study conducted in Kenya, where anemia was present in 147 patients (86.0%). In our study, fever was noted in 81.6% of patients, whereas in Rwanda, it was observed in 76% of patients [6]. Other common clinical features include lymphadenopathy in 26 patients (14.5%) and abdominal swelling in 84 (46.9%) patients, with subcutaneous nodule found in 10 (5.6%). Similarly, in Kenya, lymphadenopathy was reported in 83 (48.5%) patients, and abdominal swelling in 82 (48.0%) patients [32]. In Syria, lymphadenopathy was observed in 82.9% of patients [30].

In our study, the most common French-American-British (FAB) ALL subtype was L2 (48.0%), followed by L1 (38%), L3 (7.8%) and non-categorized cases (6.1%). This is consistent with findings from Kenya, where L1 was present in 16 patients (10.13%), L2 in 129 patients (81.65%), and non-categorized in 13 patients (8.23%) [32]. In contrast, a study from Indonesia reported that 77.5% of cases were FAB L1, 20.8% were FAB L2, and 1.7% were FAB L3 [33]. Similarly, a study from the United Kingdom found that 13% case were L2, 0.7% were L3, and 86% were L1[34]. A study conducted in Syria found L1 in 77.4%, L2 in 20.4%, and L3 in 21% [30], while a Brazilian study found L1 in 83% and L2 in 17% [35]. Our findings for L1 are comparable to those observed in Tehran, where L1 was found in 57.6% of cases, and L2 + L3 accounted for 42.4% (P > 0.05). Another study reported that L1 was present in 85–89% of cases, with L2 at 14.1% and L3 at 0.8% [36].

The results of this study indicated that 81 (45.3%) of children with ALL died during various phases of treatment. This mortality rate is higher than the 20% reported in Kosobo [18] and lower than the 71% observed in Rwanda [2]. Among these deaths, 33 (18.4%) occurred prior to induction, 14.5% during induction, and 5.6% after chemotherapy, while 9 patients (7.5%) experienced relapse. Notably, there was a 41.90% event-free survival (EFS) rate, with only 22 patients (12.3%) completing the entire treatment regimen. Our findings are lower than those of a study conducted in Cambodia, which reported a mortality rate of 34.9% [37]. A similar study in Kenya found death, relapse, and abandonment rates of 30%, 26%, and 24%, respectively, with an EFS rate of 20%. Among all recorded deaths, 80% occurred in patients who had relapsed, while only 20% were in remission [35]. Furthermore, Research from Egypt indicated relapse rates of 12% to 20% and a mortality rate of 23% [23,36]. In other study, relapse was observed in 27% of patients who had achieved remission, contributing to elevated mortality rates and a low overall survival (OS) rate at five years. Most patients who relapsed did so shortly after achieving remission [23]. Refusal and abandonment of treatment are leading causes of treatment failure [38,39].

Different studies have shown varying patient outcomes across countries. In Nicaragua, 7% of patients died during induction, and 9% abandoned treatment, with 5-year and ten-year EFS rates of 38.1% and 36.6% respectively, and OS rates of 48.0% and 39.6% [37]. In Brazil, treatment mortality varied widely from 32% to 63%, while Indonesia reported a high death rate of 60.5%. In contrast, a Central American study showed much better results: only 3.0% of patients died during induction, with just 2.7% abandoning treatment and only 1.1% developing resistant ALL. Additionally, 93.2% achieved complete remission, with deaths and treatment abandonment during the first complete remission period at only 2.7% and 7.0%, respectively [40].

Global variability in 6-mercaptopurine 6-MP-induced toxicity can be attributed to factors such as patient characteristics, follow-up periods, genetic variations, and differences in dosing and adjustment protocols [40]. This study observed a higher incidence of grade 4 neutropenia (52.2%) which is consistent with similar studies from China (47%) [41] and Thailand (47%) [42]. Swedish and Korean studies reported even higher rates [43,44]. Additionally, this study identified increased rates of 6-MP interruption and neutropenic fever compared to earlier reports [1], although some studies documented higher treatment interruptions [5]. The most common side effects reported were fever or flu-like symptoms (81.8%), itching or skin rash (68.2%), decreased appetite (50%), and behavior changes (63.6%). However, a study from Indonesia found behavior changes to be the most frequent side effect, followed by increased appetite and infections. The higher incidence of fever or flu-like symptoms in this study may be related to hematotoxicity [1]. Neutropenia was the primary cause of death-related treatment complications, often exacerbated by infections that led to life-threatening conditions requiring further treatment. This may have contributed to delays in therapy during the induction phase [45].

In our study, the leading cause of mortality was identified as treatment abandonment, often attributed to financial constraints, inadequate healthcare facilities, and misconceptions among healthcare providers. These factors contribute to delays in seeking medical attention and increase the risk of severe infections. Various studies have indicated that the predominant reasons for abandonment include financial limitations (34.5% in one study and 59.4% in another), [57], misconceptions regarding the curability of conditions (20% in one study and 22.9% in another), [57], poor overall health status of the child (15%), lack of improvement in condition (13%), and refusal to donate blood (3%). A significant number of fatalities were directly associated with infections [23].

Malaria infection complicates the treatment of ALL, increasing the risk of severe infections. Conversely, patients who tested negative for malaria were more likely to exhibit a favorable survival trajectory due to the absence of malaria-related complications. Central nervous system (CNS) involvement at baseline was notably prevalent in this study and was associated with adverse outcomes. This finding aligns with observations from high-income countries, where CNS involvement is recognized as a negative prognostic indicator in pediatric ALL [46]. The incidence of CNS disease at presentation in our study was significantly higher than that reported in high-income countries, where it typically occurs in less than 10% of cases [47]. This discrepancy may be attributed to chance, delayed disease presentation, or differences in biological factors; however, further studies are needed to investigate this issue in greater detail.

A high initial leukocyte count is a critical factor in therapy risk stratification and is associated with a worse prognosis. High-risk HR patients experience lower overall survival (OS) and EFS due to both the poorer prognosis associated with their condition and the more intensive therapy required for treatment [46]. In our study, 63 patients (48.6%) were classified as HR, while 51.4% were classified as SR. This is comparable to the HR rate of 51.4% reported in Addis Ababa (51.4%) and other SR classifications [5], in Cambodia, 57% were classified as HR and 43% as SR [37], while in Rwanda, 14.29% were SR, 21.43% were HR, 4.76% were very HR, and 59.52% were unclassifiable [6]. The highest mortality rates were observed in the HR and very high-risk groups (p < 0.001), consistent with other studies [48].

Factors contributing to the longer duration of the induction phase included infections. Inadequate clearance of leukemic cells during induction may increase the risk of induction failure and lead to a higher likelihood of relapse and severe complications [49]. However, our study shows that 10% of patients experienced relapse, which is Comparable to the study conducted in Kosobo and in Egypt, where the relapse rate was 11% [16], and 27% [23] respectively. Our study found a 7.5% induction failure rate and a 10.1% relapse rate, likely due to the reliance on morphological examination at the end of the induction phase. This method remains crucial for assessing ALL therapy in many developing countries with limited minimal residual disease (MRD) facilities. A prolonged induction phase can negatively impact prognosis and treatment outcomes, increasing the risk of complications [50]. Similar findings were reported in a study conducted in India [51], as well as in studies conducted in other countries [45,52].

In a multivariate analysis, several significant factors were identified as contributing to mortality and poor prognosis, including age, rural residency, T-cell ALL, sepsis, elevated LDH levels, relapsed ALL patients, low platelet counts, malaria infection and induction failure (all p < 0.05). These findings are consistent with numerous studies conducted in various countries, which have reported similar associations between these factors and adverse outcomes in ALL patients [45,49,52,53].

Future prospective cohort studies should investigate factors that may influence the survival of patients with ALL in LMICs. Key factors to assess include socioeconomic status, healthcare providers’ perceptions, inadequate medical facilities, parental education, family attitudes toward the disease, and the distance between patients’ homes, healthcare facilities, and pediatric oncology centers. Understanding these variables is critical for developing targeted interventions to improve treatment outcomes and survival rates in these regions.

## Conclusion

This study observed a high mortality rate among children with ALL, despite advancements in treatment. Key risk factors for death included the absence of bone marrow remission after the induction phase; age over 10 years, elevated LDH levels, and sepsis. Additionally, low platelet counts, T-cell ALL, and malaria infection were significantly associated with increased mortality risk. To improve survival rates, it is essential to address these risk factors through targeted interventions. Furthermore, optimizing treatment regimens, minimizing delays in diagnosis and treatment initiation, and providing personalized care based on individual genetic and clinical profiles are critical steps toward enhancing outcomes for ALL patients.

## Abbreviation:

ALL: Acute lymphoblastic leukemia
TPMT: Thiopurine mythyltransferase
ADR: Adverse drug reaction
EFDA: Ethiopian Food and Drug Administration
HCPs: Healthcare professionals
KAP: Knowledge, Attitude, and Practice
UoGCSH: University of Gondar Comprehensive Specialized Hospital
AAU: Addis Ababa University
AL: Acute Leukemia
AML: Acute Myeloid Leukemia
BM: Bone Marrow
CNS: Central Nervous System
EFS: Event free survival
FAB: French-American-British
Hgb: Hemoglobin
LDH: Lactate Dehydrogenase
SNNPR: Southern Nations Nationalities and Peoples Region
TASH: Tikur Anbessa Specialized Hospital
WHO: World Health Organization
